# An Unusual Presentation of Posterior Globe Rupture Detected During Pars Plana Vitrectomy

**DOI:** 10.7759/cureus.59564

**Published:** 2024-05-03

**Authors:** Mohd Faizal Zokri, Ainal Adlin Naffi, Mushawiahti Mustapha, Othmaliza Othman

**Affiliations:** 1 Ophthalmology, University Kebangsaan Malaysia Medical Center, Kuala Lumpur, MYS

**Keywords:** posterior globe rupture, vitrectomy, retinal detachment, eye foreign bodies, penetrating eye injuries

## Abstract

A 54-year-old gentleman presented with a history of poor vision in the right eye for three months and a prior forgotten trauma. The anterior segment was normal. He was diagnosed with subtotal bullous rhegmatogenous retinal detachment (RRD), but no apparent tear was observed. Vitrectomy commenced, and upon exploration, there was a posterior globe rupture with retinal and vitreous incarceration. The scleral wound was sutured with heavy liquid in situ. Orbital imaging post-surgery revealed the presence of an intraorbital foreign body. This is a peculiar presentation of posterior globe rupture, as it was unperceived by the patient, and the slit lamp examination conducted indicated no clinical evidence. Identifying posterior globe rupture remains a challenge that necessitates a high index of suspicion and appropriate management.

## Introduction

Open globe injury (OGI) is a leading cause of monocular blindness that continues to pose a management quandary. The standard practice worldwide is to restore the structural integrity of the globe by primary repair at the earliest encounter [1]. Meticulous evaluation should be performed in the presence of an intraorbital foreign body (IOrFB) to determine whether there is concurrent retinal damage and if immediate surgical intervention is necessary. In this context, we report a case of successful management of an occult posterior globe rupture leading to traumatic rhegmatogenous retinal detachment (RRD) as a sequela of a missed penetrating ocular injury associated with an IOrFB.

This case report was presented as a Free Paper at the Euretina 2021 and a video presentation at the Asia-Pacific Vitreo-retina Society (APVRS) 2021, winning one of the best video awards.

## Case presentation

**Figure 1:**
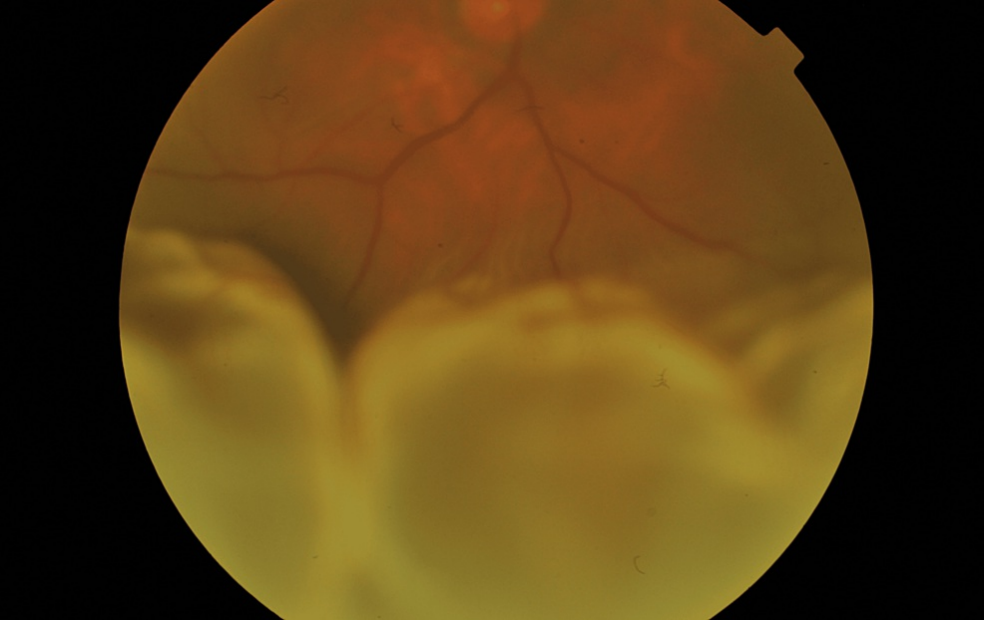
Fundus photograph of the right eye showed extensive bullous inferior rhegmatogenous retinal detachment involving the macula (macula detachment)

A 54-year-old gentleman with underlying hypertension and bilateral pseudophakia came to seek treatment for right eye blurring of vision for a three-month duration. There was no eye pain or redness. Upon further examination, his right eye visual acuity was 6/60, with a pinhole of 6/36, while his left eye visual acuity was 6/9. There was no relative afferent pupillary defect, and the extraocular movement of both eyes was normal. Both eye's anterior segments were unremarkable, having the same anterior chamber depth. The intraocular pressure (IOP) of both eyes was 12 mmHg. The vitreous was clear, with no evidence of hemorrhage. The posterior segment of the right eye showed inferior bullous RRD involving the macula. The tear was not evident on a slit-lamp examination, but the detachment configuration suggested a retinal tear above the horizontal meridian (Figure 1). The presence of extensive peripheral posterior capsular opacity further complicated the search for retinal tears preoperatively. There was minimal tobacco dusting with proliferative vitreoretinopathy grade B.

**Figure 2:**
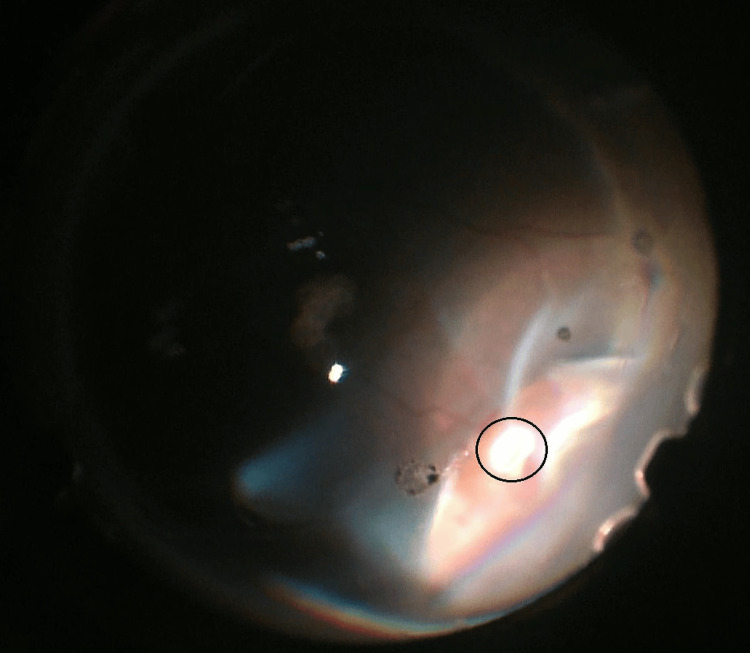
Large retinal tear with incarcerated vitreous (black circled).

Pars plana vitrectomy was performed. During core vitrectomy, there was a sudden snap (momentary collapse) of the eyeball. Concurrently, the eyeball became soft, and there was an area of choroidal collapsing. Further evaluation revealed a vitreous wick that appeared to track posteriorly toward the sclera. Adjacent to it was a large round retinal tear (Figure 2). Despite raising the infusion bottle, the eyeball remained soft. On further internal search, we found an area of suspicious posterior scleral tear at 11 o'clock with adjacent pigmentation around the end of the vitreous track. In preparation for external exploration, the eyeball was filled with heavy liquid (perfluorocarbon heavy liquids ophtha futur deca) to flatten the bullous detached retina and avoid further collapsing.

**Figure 3:**
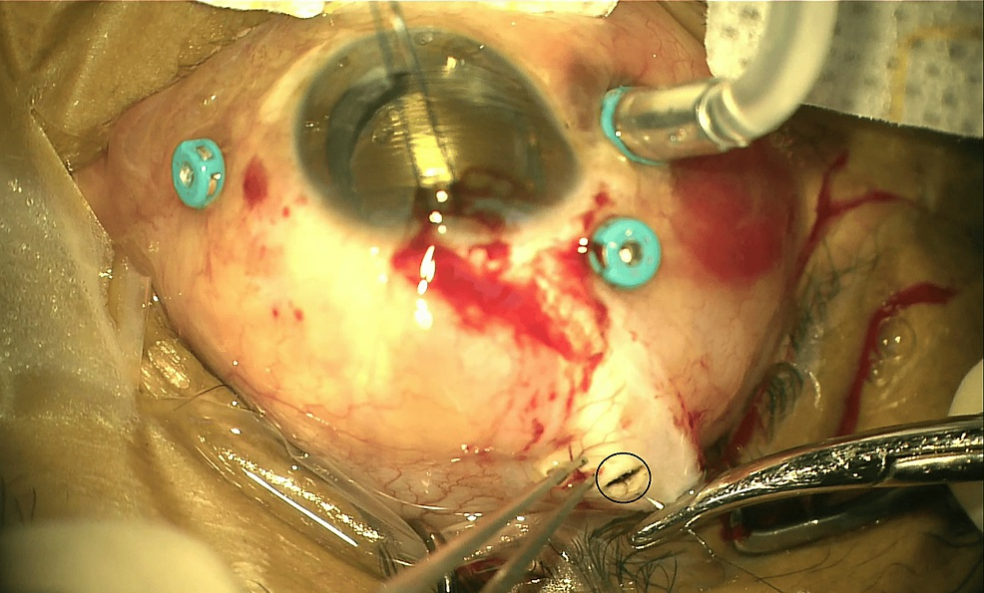
External image of full-thickness linear scleral laceration (measuring 4 mm) running horizontally at the superotemporal region, 11 mm from the limbus (black circled).

The conjunctiva was dissected at the suspected quadrant (superotemporal), and a bridle suture was placed at the limbus to rotate the eyeball safely, thereby ensuring no pressure was exerted on the globe during rotation and manipulation. Inspection of the superotemporal sclera revealed an area of linear laceration wound (4 mm in size) running horizontally at the equator about 11 mm from the limbus (Figure 3). The laceration was sutured with 8-0 nylon. After complete suturing of the globe, the eyeball turgor was restored, and vitrectomy recommenced safely.

**Figure 4:**
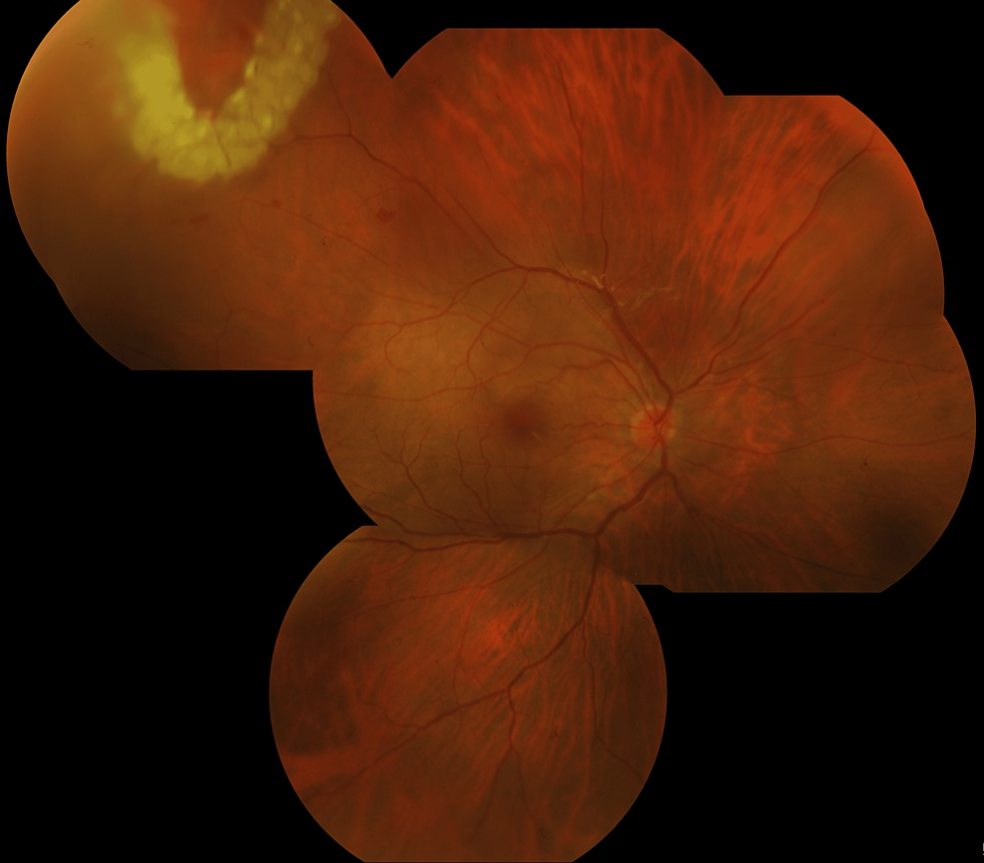
Fundus photograph of right eye postoperatively: Silicone oil filled showing flat retina with barricade laser marks around the tear

The surgery was completed with complete shaving of the peripheral vitreous. A barricade laser around the retinal tear was performed using heavy liquid. Eventually, silicone oil 1,000 centistoke was used as an endotamponade agent (Figure 4).

**Figure 5:**
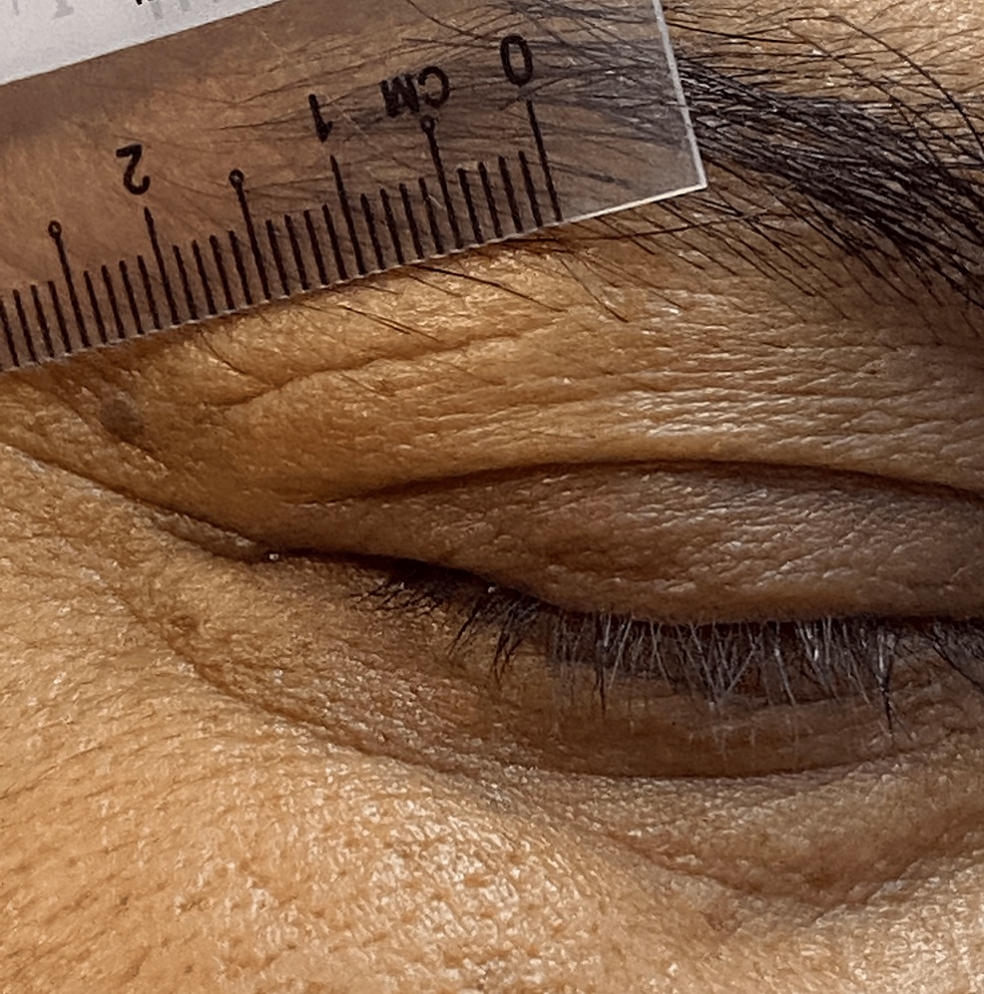
Linear healed laceration wound measuring 2 cm in length underneath the eyebrow.

**Figure 6:**
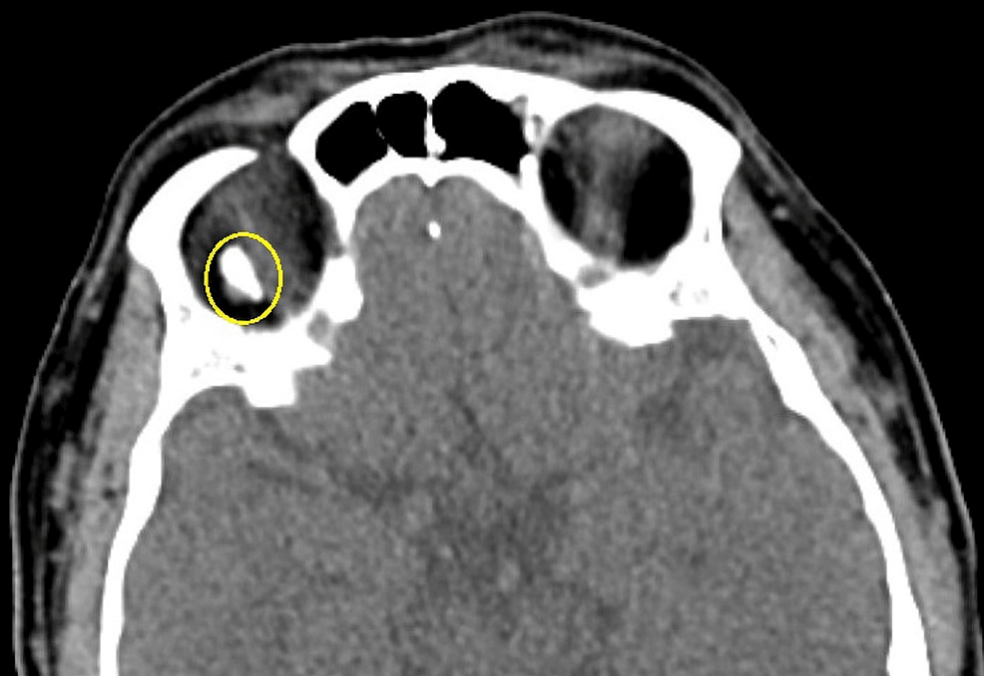
Axial view of contrast-enhanced computed tomography orbit scan showing a hyperdense foreign material situated in intraconal space (yellow circled) without surrounding tissue reaction and not encapsulated.

While questioning the patient, he vaguely remembered an episode of trivial right eye injury while riding a motorbike three to six months before the presentation. However, he could not describe in detail the nature of the injury. Upon further inspection, a fine linear healed wound over the right upper lid suggested a previous penetrating injury (Figure 5). The location of the wound within the upper lid crease corresponds to the position of the scleral perforation area underneath. Computed tomography imaging done postoperatively revealed the presence of foreign material within the intraconal space temporally (Figure 6). The intraorbital foreign material is left untouched as it does not cause surrounding tissue reactions. The patient is scheduled for closed monitoring with final visual acuity of 6/24 pin hole 6/24.

## Discussion

Occult posterior globe rupture is a traumatic dehiscence of the sclera at or posterior to the rectus muscle insertions without a visible eye wall defect, identified by a slit lamp or fundus examination because of vitreous hemorrhage [2]. Diagnosis remains challenging in this case, as it lacks several cardinal signs of occult posterior globe rupture. The most important sign of posterior scleral rupture is a posteriorly displaced plateau iris, with the anterior chamber deepening [3], which was absent in this case. In this case, the normal IOP can be misleading, as ocular dehiscence may be sealed by the vitreous, other intraocular tissue, and blood clots [3].

Furthermore, there were no apparent clinical signs of ocular trauma (absence of conjunctiva or cornea laceration) or subconjunctival hemorrhage, and the intraocular lens was perfectly intact and stable. In addition, as the patient had undergone cataract surgery with intraocular lens implantation three years prior, the presence of peripheral posterior capsular opacity obscured the full peripheral retina examination. However, the presence of proliferative vitreoretinopathy grade B suggests that RRD may be chronic in origin.

Our initial impression was pseudophakic RRD, given the presence of typical clinical signs in this patient (such as a small retinal tear with bullous retinal detachment), and the case seemed to follow the Lincoff rules [4]. Intraoperatively, we found that the patient had a posterior globe rupture, complicated with RRD, and overlying vitreous and retina incarceration, acting as tamponade or sealing on top of the RRD. When his retina was quite bullous, we had difficulty identifying the location or site of the defect because the bullous retinal detachment had covered it.

The posterior globe rupture was anteriorly located, and the posterior end of the wound was reachable and uncovered by the rectus muscles. A bridle suture was put over the corneoscleral limbus with 4-0 silk to allow better gentle manipulation of the eyeball so that the posterior sclera could be inspected and the rupture identified for further repair. These traction sutures should prevent external pressure on the sclera and the expulsion of intraocular content. The posterior scleral rupture was sutured with 8-0 nylon, as we needed good turgor and watertight of the eyeball to proceed with vitrectomy. Nylon suture was used instead of Vicryl because it is stronger and nonabsorbable. Heavy liquid needed to be placed in the eyeball to prevent the eyeball turgor from being too soft and to maintain the retina in place. This facilitated further vitrectomy and barricade laser to repair the tear internally.

Postoperatively, we proceeded with a contrast-enhanced computed tomography orbit scan, which revealed a hyperdense structure, which may suggest a metallic IOrFB, as described by Pinto et al. [5]. An IOrFB is a foreign object located inside the bony orbital walls posteriorly to the orbital septum but outside the ocular globe [6]. It can be located extraconally or intraconally [7] and may be further classified as metallic or non-metallic (organic or inorganic) in nature [8]. Objects with high velocity (usually metallic ones) penetrate deeper and commonly lodge within the posterior globe or retrobulbar structures, whereas particles with less momentum (plastic and wood) may be embedded in the anterior globe or superficial soft tissues [9].

Our patient was unaware of the poor vision in the affected eye, probably due to the nature of inferior RRD, which only affected the superior visual field and preserved his navigation and reading. Furthermore, the foreign material, which appeared to be inert and posteriorly located, did not induce an intense intraocular reaction.

The presence of a well-healed hypopigmented linear laceration wound over his right upper lid was almost invisible due to its nature, following the upper lid anatomical crease. The linear clean wound is highly suggestive of a foreign body that is sharp in nature. There are three overlapping stages of wound healing: inflammation, proliferation, and maturation/remodeling [10]. The time taken for scar maturation is between six months and one year [10]. Therefore, in our case, the incident most likely happened months before the presentation. Wounds in the forehead, antihelix, and eyelids areas heal with secondary intention- resulting in flat hypopigmented scars, which rarely require surgical repair [11].

Based on Pieramici et al.'s [12] OGI classification, posterior globe injuries occur in Zone III, which involves the sclera and extends farther than 5 mm posterior to the limbus. The incidence of retinal detachment in OGI has been described to be as high as 29% [10]. The presence of a higher zone of injury (zone III) at the time of presentation is associated with an increased risk of retinal detachment. As a preventive measure, the patient should wear a proper helmet with protection for both eyes while riding a motorbike, as Ramli et al. [13] demonstrated that improper helmet use and not having a fastened helmet increases the risk of facial injuries.

## Conclusions

Diagnosis of occult posterior globe rupture leading to traumatic RRD associated with an IOrFB remains a challenge. Thus, a high index of suspicion is warranted. A detailed history, ocular examination, and imaging investigation are needed to determine the possible etiology before initiating surgery. Some patients may remain asymptomatic until a complication or sequelae of retinal detachment occurs. Furthermore, as a prevention step, proper helmet usage, with a proper visor to protect the eyes, is crucial to prevent any incidental IOrFB eye trauma that can subsequently lead to detrimental sequelae.
